# Supplementary material for: Meta-Analysis and Gene Set Enrichment Relative to ER Status Reveal Elevated Activity of MYC and E2F in the “Basal” Breast Cancer Subgroup
Source: PLoS One. 2009 Mar 9;4(3):e4710. doi: 10.1371/journal.pone.0004710 (PMC2650420; doi:10.1371/journal.pone.0004710)
Supplement: Dataset S1 — GSEA results from first screen. The file contains a zipped archive of the HTML results of the second GSEA screen. Reports for enriched or depleted datasets may be accessed from the “index.html” link within the my_analysis.GseaPreranked.1219975950625.rpt.zip ZIP archive. Enrichment plots are available for each of the 50 most enriched and depleted gene sets. (13.32 MB ZIP) [file pone.0004710.s001.zip › my_analysis.GseaPreranked.1219975950625.rpt.zip!//FLAGELLAR_ASSEMBLY.html]

Details for gene set FLAGELLAR\_ASSEMBLY[GSEA]

|  || Dataset | zSco.ERneg.to.ERpos\_collapsed |
| Phenotype | NoPhenotypeAvailable |
| Upregulated in class | na\_neg |
| GeneSet | FLAGELLAR\_ASSEMBLY |
| Enrichment Score (ES) | -0.55278337 |
| Normalized Enrichment Score (NES) | -1.7712648 |
| Nominal p-value | 0.005194805 |
| FDR q-value | 0.04408343 |
| FWER p-Value | 0.845 |
Table: GSEA Results Summary

  

Fig 1: Enrichment plot: FLAGELLAR\_ASSEMBLY      
 Profile of the Running ES Score & Positions of GeneSet Members on the Rank Ordered List

  

| PROBE | GENE SYMBOL | GENE\_TITLE | RANK IN GENE LIST | RANK METRIC SCORE | RUNNING ES | CORE ENRICHMENT || 1 | ATP6V1F |  |  | 3030 | 1.919 | -0.1727 | No |
| 2 | ATP5O |  |  | 3901 | 1.554 | -0.1935 | No |
| 3 | ATP6V0A4 |  |  | 4096 | 1.475 | -0.1657 | No |
| 4 | ATP6V1D |  |  | 5604 | 0.943 | -0.2520 | No |
| 5 | ATP6V0E |  |  | 5776 | 0.888 | -0.2393 | No |
| 6 | ATP6V1B2 |  |  | 8280 | 0.007 | -0.4273 | No |
| 7 | ATP6V1C1 |  |  | 8513 | -0.084 | -0.4424 | No |
| 8 | ATP6V1A |  |  | 9715 | -0.676 | -0.5132 | No |
| 9 | ATP6V0A1 |  |  | 9795 | -0.721 | -0.4985 | No |
| 10 | ATP6V0B |  |  | 10498 | -1.161 | -0.5179 | Yes |
| 11 | ATP6V1B1 |  |  | 10963 | -1.541 | -0.5085 | Yes |
| 12 | ATP6V1G2 |  |  | 11197 | -1.742 | -0.4761 | Yes |
| 13 | ATP6V1H |  |  | 11339 | -1.867 | -0.4331 | Yes |
| 14 | ATP5E |  |  | 11436 | -1.975 | -0.3836 | Yes |
| 15 | ATP6V0D1 |  |  | 11770 | -2.294 | -0.3428 | Yes |
| 16 | ATP6V1E1 |  |  | 11864 | -2.389 | -0.2812 | Yes |
| 17 | ATP6V0C |  |  | 12506 | -3.271 | -0.2354 | Yes |
| 18 | ATP6V1G1 |  |  | 12838 | -3.971 | -0.1463 | Yes |
| 19 | ATP6AP1 |  |  | 13285 | -6.357 | 0.0026 | Yes |
Table: GSEA details [plain text format]

  

Fig 2: FLAGELLAR\_ASSEMBLY: Random ES distribution      
 Gene set null distribution of ES for **FLAGELLAR\_ASSEMBLY**
